# American Asylums for the Insane
*Reports of Institutions for the Insane in the United States.1. Of the McLean Asylum, for 1851 and 1852.2. Of the Butler Hospital, for 1851 and 1852.3. Of the Retreat at Hartford, for 1851 and 1852.4. Of the Maine State Hospital, for 1851 and 1852.5. Of the New Hampshire Asylum, for 1852.6. Of the Vermont Asylum, for 1851.7. Of the Boston Hospital (Paupers), for 1849, 1850, and 1851.


**Published:** 1854-04-01

**Authors:** Pliny Earl


### AMERICAN ASYLUMS FOR THE INSANE.*

BY PLINY EARL, ESQ., M.D.

1. The report by Dr. Bell, of the McLean Asylum, for the year 1851, is, like most of its predecessors, very brief, and is mostly devoted to a description of the method by which the Cochiuate water has been introduced into the buildings of the institution. A disbeliever in the value of statistics in insanity, the Doctor makes his practice consistent with his theory by restricting these data to the simple facts—or, as he would say, "facts and opinions" of admission and condition at the time of discharge.

| | Men. | Women. | Total. |
|---|---|---|---|
| Patients admitted in course of the year . . | 89 | 79 | 168 |
| Whole number in the Asylum . . . . | 185 | 179 | 364 |
| Discharged . . . . . . . . . | 90 | 83 | 173 |
| Remaining at end of year . . . . . | 95 | 96 | 191 |
| Of those discharged, there were cured . . | 40 | 35 | 75 |
| Died . . . . . . . . . . | 15 | 14 | 29 |

"Of the deceased, eight were not under seventy years, and one was over ninety."

In the report for 1852, it is stated that "the call for room, during the past year, has far exceeded anything in our former experience. It is within bounds to say, that we have been obliged to refuse more female patients than we have received, and probably as many of both sexes as we have admitted. * * * * Our last year's experience demonstrates that another institution of the magnitude and character of this is as urgently demanded as have been any of our previous substitutes and extensions, which have, one after another, raised our aggregate from the less than seventy, whom I found on taking the charge, to the nearly three times that number we have been obliged to find a place for during the past three or four years."

* Reports of Institutions for the Insane in the United States.
1. Of the McLean Asylum, for 1851 and 1852.
2. Of the Butler Hospital, for 1851 and 1852.
3. Of the Retreat at Hartford, for 1851 and 1852.
4. Of the Maine State Hospital, for 1851 and 1852.
5. Of the New Hampshire Asylum, for 1852.
6. Of the Vermont Asylum, for 1851.
7. Of the Boston Hospital (Paupers), for 1849, 1850, and 1851.

Hence the Doctor recommends that another establishment, disconnected from the McLean Asylum, and intended for females alone, should be founded. "There are no advantages," he observes, "of which I am aware, in having the insane of both sexes in one institution of this kind, whatever may be the case in pauper establishments, or where labour is expected of the inmates. On the contrary, there are many inconveniences and disadvantages. The customary arrangement of patients of both sexes in the same place, doubtless, had its origin in the expectation that only so many sufferers could be aggregated as would be sufficient for the full employment of a single directing head. It seems not a little singular, that a custom entailing so many objections should have been continued where its original basis had ceased to exist."

| | Men. | Women. | Total. |
|---|---|---|---|
| Patients admitted in the course of the year . | | | 145 |
| Discharged . . . . . . . . | | | 135 |
| Remaining at the end of the year . . . | | | 201 |
| Of those discharged, there were cured . . | 37 | 35 | 72 |
| Died . . . . . . . . . . | 11 | 4 | 15 |
| Admitted from 1837 to 1852, inclusive . . | | | 2339 |
| Recovered " " " . . | | | 1173 |
| Died " " " . . | | | 271 |
| 2. The number of patients in the Butler Hospital, on the 31st of December, 1850, was . | 50 | 63 | 113 |
| Admitted in the course of 1851 . . . . | 33 | 35 | 68 |
| Whole number . . . . . . . . | 83 | 98 | 181 |
| Discharged . . . . . . . . . | 22 | 32 | 54 |
| Remaining, December 31, 1851 . . . . | 61 | 66 | 127 |
| Of those discharged, there were cured . . | 8 | 18 | 26 |
| Died . . . . . . . . . . . | 7 | 9 | 16 |

*Causes of death.*—Chronic mania 8, "Bell's disease" (typho-mania?) 4, paralytic affections 2, meningitis 1, extensive pulmonary and intestinal disease 1.

The returns of the United States census, for 1850, give the number of the insane in Rhode Island as 233, or 1 in 633 of the population. In 1850, the legislature of the State appointed a commissioner to inquire into the condition of the public poor, and the insane. He visited all the towns, with a single exception, and reported the number of insane as—paupers 143, all others 140, total 283, or one in 521 of the population. Dr. Ray remarks, that "guided by such information as we happen to possess in regard to the immediate question, and generally, by the amount of error such inquiries usually present, I think we may safely say that an addition of 50 per cent. would better express the actual truth. This would make the number of insane 420, or one to every 351 of the inhabitants.

The increasing prevalence of insanity, which is implied by these statistics, induces Dr. Ray to a discussion of one of the most productive causes of this fearful malady—defective or perverted education. "The gross neglect," he observes, "of the moral powers, those which guide the passions and determine the notions, is the crowning defect of the education of our times, ruinous in its consequences to the health, both of body and mind." He recognises "the home" as the place in which children should be taught "to acquire the power of governing passion and resisting the impulses of the lower appetites, of discerning the nicer shades of right and wrong, of sacrificing self to the call of benevolence or duty, and, amid trial and change, steadily keeping in view the great purposes of life. The time has never been," he continues, "when this kind of training, in its highest condition, was very general in our country; but

I submit, as a matter of fact, whether, imperfect as it has been, it has not greatly declined during the last few generations? Unquestionably, at one time, the domestic rule was needlessly rigid and disagreeable, and led to an asceticism of manners equally prejudicial to the mental health and the moral welfare. * * * * * At present, however, we have little to fear from this source, the danger all lying in the opposite direction. The asceticism of our ancestors was infinitely less injurious than the licence which characterizes the domestic training of their descendants. How many of this generation complete their childhood, scarcely feeling the dominion of any will but their own, and obeying no higher law than the caprice of the moment. * * * * * The legitimate result of these defects in the education of our time is, that finally the ordinary virtues of life are degraded to a very subordinate rank. Patient and persevering industry, with its slow and moderate rewards, honest frugality, and a temperance that restrains every excess, frequent and faithful self-examination, clear and well-digested views of duty, become distasteful to the mind which can breathe only an atmosphere of excitement, craving stimulus that rapidly consumes its energies and destroys that elasticity which enables it to arise from every pressure with new vigour and increased powers of endurance. * * * * * The conclusion of the whole matter is, that insanity must necessarily increase in our community until the moral faculties shall be subjected to a higher culture, both in the school and the family."

Report for 1852:—

| | Men. | Women. | Total. |
|---|---|---|---|
| Patients, January 1, 1852 . . . . . | 61 | 66 | 127 |
| Admitted in course of the year . . . . | 39 | 62 | 101 |
| Whole number . . . . . . . . | 100 | 128 | 228 |
| Discharged . . . . . . . . . | 36 | 50 | 86 |
| Remaining December 31, 1852 . . . . | 64 | 78 | 142 |
| Of those discharged, there were cured . . | | | 30 |
| Died . . . . . . . . . . . | | | 15 |

*Causes of death.*—Acute mania 2, chronic mania 5, Bell's disease 3, general paralysis 1, apoplexy 1, phthisis 1, heart affection 1, suicide 1.

"As usual, several deaths occurred within a day or two after admission, from that very fatal form of cerebral disease, which, under the various names of 'meningitis,' 'brain fever,' 'phrenitis,' and 'Bell's disease' (and he might have added 'typho-mania,' the most common of all), has now become very common in our establishments for the insane."

This paragraph is quoted for the purpose of calling the attention of the general practitioner to the peculiar form of mental derangement therein mentioned. It is becoming quite common, particularly in large cities, and it is a form in which the unpractised physician is almost certain to pursue a deleterious course of treatment. I once had two cases sent to me by one physician in the course of a few days, in both of which liberal venesection had been practised. They died, as I believe such patients invariably do, after general bloodletting. It appears to be acknowledged, by all who have had experience in the treatment of this special form of mania, that a fatal result can be avoided only by active stimulation. Dr. Bell's description of the disease may be found in the *Journal of Insanity*, for 1849 or 1850.

During the first five years of the operations of the Butler Hospital, 491 patients were admitted, 127 discharged cured, and 80 died. During a greater part of the year for which the report is before us, the number of patients exceeded what was originally supposed to be the utmost limit of the means of accommodation.

In this report, as in its predecessor, Dr. Ray takes up the subject of the semeiology of insanity, and handles it with that masterly power which is exhibited in all his efforts, wherever he writes seriously and in earnest.

We can only give a general idea of this valuable, interesting, and truthful essay.

"Special and particular cases of insanity," he remarks, "no doubt there are, but the immense disparity between our own and all other times, in the prevalence of this disease, can only be attributed to the peculiarities which distinguish it from all other times. The press and the rostrum, the railway and the spinning-jenny, the steam-engine and the telegraph, republican institutions and social organizations, are agencies more potent in preparing the mind for insanity than any or all those vices and casualties which exert a more immediate and striking effect. This is the price we pay for civilization, and we shall continue to pay it, until that very distant day when men will have learned the difficult lesson of using their blessings without abusing them.

"The present is an age of great mental activity all over Christendom, and especially with us. The amount of it now required for maintaining the ordinary routine of the world, would have passed all conception a century ago. Especially has this been obvious in that constantly progressive enlargement of the field of industry, whereby the attention of men has been turned to an increasing variety of pursuits. * * * * * When we consider the amount of thought that has been concerned in bringing the manufacture of a pin, or a screw, to its present state of perfection, we may have a remote conception of the amount of that kind of mental exercise which is required in creating and conducting the countless processes of human industry. * * * * *

"No single incident of civilization has contributed so much to maintain the mental activity of modern times, as the art printing. * * * * * The multiplicity of books and of readers not only evinces a degree of mental activity which, a century ago, would have been thought to be scarcely within the bounds of possibility, but much of the literature of the day is more or less directly addressed to the lower sentiments of our nature, thereby impairing that supremacy of the higher which is indispensable in a healthy, well-ordered mind. * * * * * It is accessible to every reader in the land, and a large portion of those whom it attracts will be found among the young. If any one is disposed to doubt the accuracy of the fact, or the magnitude of its evils, let him look through any asylum in the country, and there will he see many a young man, once remarkable, perhaps, for endowment and promise, presenting one of the most loathsome and hopeless forms of disease, and will learn, upon examination, that in many the evil originated chiefly in the reading of books addressed to the imagination and passions. When we consider, too, that cases of this kind seldom recover, and thus add, by accumulation, to the actual amount of insanity in the world, the fact will account for much of its recent increase.

"Much of the mental activity that characterizes our people arises from the abundant opportunities that are offered for the pursuit of wealth, and the consequent variety and novelty of the enterprises undertaken for this purpose. * * * * * The result (of fortunate speculations) all can see and admire, but few know anything of the wear and tear of mind by which it was achieved, of the laborious calculations, the anxious moments, the sleepless nights, the joy of success, the apprehension of failure. Indeed, our ways of doing business, our notions of property, our ideas of happiness, all indicate, as strongly as traits of character can, that a large portion of our fellow-citizens habitually live and move, and have their being, under an extraordinary pressure of excitement which brooks neither failure nor delay. * * * * * The cracking strain of all the faculties most concerned in the management of business, the hopes and fears, the joy and the sorrow, the anticipations of success or defeat, produce a rapid consumption of the mental energies, which strongly predisposes the mind to insanity.

"Over and above that mental activity which is excited by the ordinary pursuits of life, there prevails among us a disposition to penetrate into untrodden fields of inquiry; to construct new systems of philosophy and science; to become absorbed in themes of a special and peculiar character; and especially to speculate in whatever is strange or mysterious, whether in the natural or the spiritual world. * * * * * We question everything; we pry into everything; and, in our opinions, we bring many things to light. * * * * * Animal magnetism, biology, communications with the spiritual world, are now discussed by multitudes with a deeper interest than they ever manifest in those immutable laws of nature which, if understood and observed, would vastly enlarge the sum of human happiness. * * * * * We are naturally led to another manifestation of the mental activity of our times, especially important as being the prolific parent of many others. The intellectual education of the young, on which we are disposed to pride ourselves so highly, is more calculated to stimulate a few of the mental faculties than to produce the harmonious development with the strong and healthy condition of all. * * * * * It may make brilliant and showy men, not incapable, in fact, of producing a sensation in the world, but it will not preserve them from the seductions of fashionable systems in philosophy or morals, nor fit them for meeting the practical exigencies of life in the best possible manner. * * * * * Under a more rational training, we have a right to suppose that a multitude of subjects which now seriously engage the attention of men, with no better results than to weaken, if not destroy, every conservative principle in their minds, would never be entertained, and thus a prolific source of insanity would be avoided.

"Another mental habit of our times, strongly calculated to produce an unhealthy condition of mind, is that of concentrating the thoughts and interests upon a single idea. Whatever subject is deemed worthy of promotion, whether it be morals, politics, literature, or religion, that object is thenceforward regarded as of paramount importance, compared with which all others sink into insignificance. By the individual, it is believed to be the great question of the day, and destined, like Aaron's rod, to swallow up every other. * * * * * At last, he gets to think that there is no hope for the race beyond the pale of his little *ism* or *ology;* and in his zeal for propagating it, he is ready to ride, roughshod, over the most deliberate convictions and most cherished sentiments of his fellow-men. * * * * * This habitual confinement to a very limited sphere of thought, tends to invest the favourite idea with a false colouring, if I may so speak, which distorts its natural proportions and relations, until it finally assumes all the characters of a *delusion.* * * * * *

"Another characteristic of the present generation, deserving of notice in this connexion, is a remarkable proneness to excess and exaggeration in all its intellectual manifestations. Truth is supposed to require a high colouring to make it sufficiently impressive; while the calm, the plain, the moderate, whether in the subject-matter or the form of expression, is apt to be regarded as 'stale, flat, and unprofitable.' * * * * * High-sounding words are mistaken for depth of meaning, extravagance for intensity, and the feverish heat of a jaded fancy for the fervors of a true inspiration. * * * * * To be popular, philosophy must abound in startling theories, and challenge our strongest and dearest convictions; education must aim at apparently great results, rather than the vigorous growth and symmetrical development of the mental faculties; poetry and romance must lay bare the morbid anatomy of the heart, in order to find the real sources of moral life and the true principles of social organization. * * * * * It cannot be questioned that this fondness for the intense, whether real or mock, is unfavourable to mental health, and has contributed, in some degree, to the recent increase of insanity among us.

"Perhaps nothing is better calculated to foster the kind of mental activity in question, than the practical working of our republican institutions. * * * The political agitation, which is never at rest, around the citizen of a republic, is constantly placing before him great questions of public policy, which may be decided with little knowledge of the subject, but none the less zeal—perhaps with more. * * * * Whatever be the occasion, he feels called upon to have an opinion of his own; and if an eye to the main chance shows it to be unsafe to speak out his thoughts, then his ingenuity is exerted to conceal them by means of false issues, double-meaning, and non-committal, and the amount of mental exercise necessary for this end would suffice, half a dozen times over, for the ordinary routine of life. But the mental activity which is excited directly by free institutions is not confined to political matters. It pervades every sphere of action, every exercise of thought. The almost absolute freedom from restraint, and the independence of foreign control, even in opinions merely, lead to a certain hurry and impetuosity of the vital movements, and an impatience that seeks for results by extraordinary effort or superficial methods. * * * We rush into every strife, and take sides in every question that agitates the public mind. * * * We have no idea of any division of labour here, and think ourselves as competent to sit in judgment on questions that have accidentally been brought before the public notice, as they who have made them the study of a lifetime. If, in this way, every man is not his own doctor, or lawyer, or minister, yet he enters, with the zeal of a partisan, into every contest between rival systems of medicine, law, and divinity. * * * How different, in this respect, is the present generation from all the past, in which people were quite satisfied, in regard to certain subjects, with taking their opinions upon trust, in the belief that others might be better qualified, by education and experience, to form them than they were themselves, and thereby avoided one fertile source of that excitement and agitation which prepare the mind for insanity."

This is but a mere skeleton of the article before us; but, in the language of the article itself, it is "enough for those who are disposed to profit by the warning; and too much, probably, for the larger number, who will regard it as merely an ingenious speculation."

Near the close, Dr. Ray asks, "Whoever heard of a book on mental dietetics, or has the slightest suspicion that the health of the mind may be affected by the manner in which its exercise is managed?" This query is now satisfactorily answered, by the admirable little treatise, by Dr. Feuchtersleben, entitled "Dietetics of the Soul," originally published at Vienna, but translated and republished in London. A few copies have been imported, and found an immediate sale. Although it contains some extravagances, yet it should be read by every physician, as a certain modicum of the doctrines inculcated therein would be of essential value in general practice.

3. The report for 1851 from the Hartford Retreat gives the following statistics:—

| | Men. | Women. | Total. |
|---|---|---|---|
| Patients, April 1, 1851 | 72 | 85 | 157 |
| Admitted in course of the year | 68 | 90 | 158 |
| Whole number | 140 | 175 | 315 |
| Discharged | 52 | 82 | 134 |
| Remaining, April 1, 1852 | 88 | 93 | 181 |
| Of those discharged there were cured | 26 | 42 | 68 |
| Died | 9 | 13 | 22 |

*Causes of death.*—Consumption 2, chronic inflammation of the intestines 2, cancer 1, apoplexy 3, general paralysis 2, paralysis 5, dysentery 4, exhaustion 2, general debility 1.

Nine years ago the daily average number of patients was 84; during the year covered by this report it was 168.

The following remarks by Dr. Butler will apply with equal truth to any good asylum, and the ideas therein contained should always be considered in making an estimate of the utility of institutions for the insane: "The benefits conferred by the institution will not be correctly appreciated, if estimated alone by the number of those discharged as recovered. Among those who leave us as more or less improved, or whose mental state is reported as stationary, are many who have received benefits little less in importance to themselves and their friends, than that of restoration to sanity. These take with them, besides an improvement of their general health, greater ability to take proper care of themselves, to control their impulses, and to make a better use of their remaining powers of body and of mind. If patients cannot be restored to reason, it is something to have acquired habits of cleanlinesss and decency, of peacefulness and industry."

The custom, so common in Continental Europe, of connecting with the institution for the insane, chaplains, whose duty it is to visit the patients daily, has met with but little favour upon this side of the Atlantic, and the Retreat is the only asylum at which it has been adopted. The numerous and grave objections to the plan have there been overcome or rendered nugatory by the rare qualifications of the incumbent. Were all clergymen *fac-similes* of the late Thomas H. Gallaudet; did they understand human nature, psychology, and insanity as well as he; and had they that peculiar natural adaptation to the place which no individual training or effort can attain, the custom might be generally followed, and, undoubtedly, with material benefit. "His equanimity and calmness," says Dr. Butler, "checked the unduly excited; his suavity and quiet dignity calmed the turbulent; his kindness, cheerfulness, and wit, with his ready repartee, cheered and amused the desponding; while his rare conversational powers, and his fund of anecdote, and of general and useful knowledge, made him the welcome companion of all. His aptness of illustration, the happy manner in which he applied practical religious truth to the varying circumstances of the different patients, together with his quick perception of individual peculiarities, gave him ready access to every mind, especially to that class of religious monomaniacs who are difficult of approach, and whose minds appear most obstinately closed against right and natural views. * * * He seemed to bring sunlight with him into our household, and he left its cheering influences in every heart."

The report for 1852 is from the pen of Dr. E. H. Hunt, who acted as physician to the Retreat during the absence, on a voyage to Europe, of Dr. Butler:—

| | Men. | Women. | Total. |
|---|---|---|---|
| Patients, April 1, 1852 | 88 | 93 | 181 |
| Admitted in course of the year | 66 | 74 | 140 |
| Whole number | 154 | 167 | 321 |
| Discharged | 74 | 77 | 151 |
| Remaining, April 1, 1853 | 80 | 90 | 170 |
| Of those discharged, there were cured | 32 | 32 | 64 |
| Died | 11 | 10 | 21 |
| Whole number, since opening of the Retreat | | | 2318 |
| Discharged, cured | | | 1267 |
| Died | | | 243 |

It appears from this report, that there are no statute laws in regard to the confinement of patients in the Retreat. Dr. Hunt very justly remarks, that "the security of the public demands that some simple and readily accessible means of approximately determining the fact of insanity, by means of a careful investigation of each case by some independent and impartial tribunal, should be provided by legislation, and that its requirements, in all cases, be complied with."

Dr. Hunt suggests that, in order to "render the institution worthy, at least, of the entire confidence of even the most timid and exacting," there shall be "a regular monthly visit of two of the members of this board, who shall make a thorough examination of every part of the institution, and learn the reason for every apparent indication of severity or neglect." Now, the Retreat, among its immediate officers, has a chaplain, who, by virtue of his sacred profession, ought not to brook the least maltreatment of the patients; it has its "Board of Managers," and a "Medical Board," both of which, as appears by the report, make "frequent official and unofficial visits;" and it has a "Visiting Committee of Ladies," numbering seven persons. If all these guards are insufficient to prevent the evasion of abuses, it would seem that prevention is impossible. They are enough, at least, to render the labours of the superintendent sufficiently onerous; and, if the proposed committee be appointed, we would advise him, unless he wishes soon to be compelled again to flee to Europe in search of health, to suggest still another committee—a "Resident Committee for the Reception of Committees." That which Dr. Bell very properly terms "*the tittle-tattle*," in regard to alleged abuses at the asylums, constitutes a portion of the mental pabulum of certain classes of the people, and can no more be suppressed by additional efforts for the prevention of any real cause in which they might originate, than the delusions of an insane person can be removed by assuring him that they are erroneous and absurd.

4. The report of the Maine Insane Hospital embraces a period of twenty months, from the 31st of March, 1851, to the 30th of November, 1852.

| | Men. | Women. | Total. |
|---|---|---|---|
| Patients at the commencement of the period . | 34 | 24 | 58 |
| Admitted since . . . . . . . . | 64 | 35 | 99 |
| Whole number . . . . . . . | 98 | 59 | 157 |
| Discharged . . . . . . . . . | 47 | 25 | 72 |
| Remaining at the close of the period . . | 51 | 34 | 85 |
| Of those discharged, there were cured . . | 22 | 34 | 34 |
| Died . . . . . . . . . . | 8 | 1 | 9 |

"1 died with pneumonia, 3 with general paralysis, 1 with scrofula, 1 with malignant sore throat, 1 with epilepsy, 1 with inflammation of the liver, and 1 with consumption."

Since the institution was opened it has received 115 suicidal patients, 61 males and 54 females. Only 2 of these, and those both males, have committed suicide while in the hospital, though several have done so after being removed therefrom. Of homicidal patients there have been 69, 53 males and 16 females. "No accident from any of these has ever occurred." "The suicidal form of insanity is as likely to recover as any other form, but the homicidal much more rarely recovers." "There have been 20 who had both suicidal and homicidal propensities, 11 males and 9 females."

Of patients inheriting a predisposition to insanity, "337 have enjoyed the benefits of the hospital and been discharged. 148 of them went home cured; a proportion nearly equal to that which obtains among those who do not inherit the disease."

Dr. Harlow thus writes of the practical operation of *that* "Maine Law" which provides that all persons charged with crime, and alleged to be insane, shall be removed to the Insane Hospital for the purpose of testing the validity of the allegation: "We have had 5 such cases within the last twenty months, sent here by order of the court. They were all males. Three of them were charged with the crime of *arson*, 1 with *larceny*, and 1 with *assault with intent to kill*. Three proved to be insane beyond a doubt. The other two were brothers, one aged seventeen, the other ten, and both charged with the crime of *arson*.

"In the case of the elder boy, so much doubt existed in relation to the presence of insanity, that he was removed from the hospital soon after we reported him to the court. The younger boy is of diminutive size, physically slender, strongly marked with the nervous temperament, quite active and irritable, and has rather a wild, peculiar expression of the eye, and the impediment of stammering. He is unlike any other boy we have ever seen, an enigma of no easy solution. He possesses a good memory, an uncommon observation, great inquisitiveness, acute perception, strong affection, emotion, and feeling, little or no judgment, and a will which brooks restraint with great difficulty. He is naturally far from being malicious, but possesses kind and tender feelings towards all, except when under the influence of passion. He is forward, fearless, and bold. He is a creature of impulse; and here, we consider, lies the secret of the whole matter. Impulse, if we may so speak, usurped all power, and impelled him, in the absence of judgment and all conscience, without motive or thought, to commit the crime with which he is charged. We could not consider him in any other light than as an irresponsible boy, as not accountable for the acts which he committed, on the ground of *an undeveloped judgment and a want of conscience*."

5. Dr. McFarland having resigned the superintendence of the New Hampshire Asylum, his place has been filled by the appointment of Dr. John E. Tyler, the author of the report now before us.

| | Men. | Women. | Total. |
|---|---|---|---|
| Patients on the 31st of May, 1852 | 63 | 55 | 118 |
| Admitted in course of the year | 68 | 64 | 132 |
| Whole number | 131 | 119 | 250 |
| Discharged | 61 | 46 | 107 |
| Remaining, May 31, 1853 | 70 | 73 | 143 |
| Of those discharged, there were cured | 41 | 22 | 63 |
| Died | 5 | 3 | 8 |

*Causes of death.*—Chronic mania 3, consumption 2, exhaustion 2, suicide 1.

From the opening of the asylum, in 1848, 1058 patients have been received, 434 discharged recovered, and 92 have died.

The report is brief, and does not touch upon any important subject which has not heretofore been fully discussed in these "notices."

6. The report of Dr. Rockwell, of the Vermont Asylum, is limited to three pages.

| | Men. | Women. | Total. |
|---|---|---|---|
| Patients on the 1st of August, 1851 | 173 | 155 | 328 |
| Admitted in course of the year | 63 | 74 | 137 |
| Whole number | 236 | 229 | 465 |
| Discharged | 67 | 63 | 130 |
| Remaining, August 1, 1851 | 169 | 166 | 335 |
| Of those discharged, there were cured | | | 73 |
| Died | | | 35 |
| Admitted since the opening of the asylum | | | 1746 |
| Discharged cured | | | 818 |

A severe form of dysentery prevailed, chiefly in the months of August and September (1850). 93 patients were attacked by this disease, of whom

16 died. Nearly all our attendants and assistants were attacked by the same disease, all of whom recovered.

In treating of the caution necessary to be observed in regard to the removal of persons to the asylum, Dr. Rockwell makes the subjoined remarks, which coincide with the opinions which we long since formed and expressed in regard to the class of cases in question.

"There is one class of cases, especially, which are frequently sent too early to a lunatic asylum, I mean that of puerperal cases. We have repeatedly had women brought to the asylum in less than two weeks from their accouchement. Some of them have recovered very soon, but would, probably, have recovered as well had they remained at home. Others have died, apparently from exhaustion, who might have recovered had it not been for the exposure and fatigue of the journey."

7. The Boston Lunatic Hospital was opened in 1839, and the report of 1849, by Dr. Stedman, contains the principal data in regard to the movement of its population, during the first decennium of its existence.

| | Men. | Women. | Total. |
|---|---|---|---|
| Whole number of patients . . . . . | 313 | 298 | 611 |
| Discharged . . . . . . . . . | 232 | 175 | 407 |
| Cured . . . . . . . . . | | | 180 |
| Died . . . . . . . . . . | | | 129 |
| Remaining . . . . . . . . . | | | 204 |

Single 329, married 211, widowed 61, unknown 10.

*Causes of death.*—Consumption 26, marasmus 13, general paralysis 11, epilepsy 10, dysentery 10, Asiatic cholera 10, general debility 9, diseases of heart 8, hemiplegia 5, exhaustion 4, chronic diarrhœa 4, suicide 3, chronic inflammation of brain 2, erysipelas 2, variola, inflammation of stomach and intestines, inflammation of intestines, fungus hæmatodes, typhoid fever, scrofula, cholera morbus, pleuro-pneumonia, tubercular peritonitis, cancer, purpura, and wound of an artery, 1 each.

"During the last year, and that preceding, dysentery prevailed epidemically, and very severely. Erysipelas has been of frequent occurrence among us. The Asiatic cholera was not so terrific in its ravages here as we had anticipated. On its first irruption, provision was made for such patients as might be attacked by it, by converting our bowling-alley building into a cholera hospital. Great care was also taken in properly ventilating the halls and rooms of the main hospital, in regulating the diet, and in watching and arresting the very first movements towards this affection. Numerous inmates were seized with diarrhœa during this epidemic, and had it not been for the timely and successful attempts made to check this apparently premonitory symptom, cholera would not have left this household so free from its ravages as it did. The number of those prostrated with true cholera was 16. Of these, 10 died; all of whom had been insane for too long a period to allow of any expectation of recovery from their mental disease."

In the course of the year one of the patients stabbed another in the leg, with a knife which he procured in the dining-room. The hemorrhage was arrested by tying the femoral artery, and, twelve days afterwards, secondary hemorrhage by tying the external iliac. Death, however, ensued, twenty-five days subsequent to the primary wound. The investigation by the grand-jury established the fact that the attendant upon the patient who committed the outrage had neglected to follow the printed rules of the house, obedience to which would have prevented the accident.

In a case of prolonged abstinence from food, Dr. Stedman administered chloroform, and, while the patient was under its influence, "nourishing liquids were readily swallowed." "I have since," he remarks, "resorted to the same treatment in other cases, with a like beneficial result. My belief now is, that in anæsthetic agents we have a perfect preventive of self-destruction from starvation, in those cases, at least, where there is no organic lesion of the stomach."

From the report for 1850.

| | Men. | Women. | Total. |
|---|---|---|---|
| Number of patients, November 30, 1849 | 80 | 123 | 203 |
| Admitted in course of the year | 27 | 46 | 73 |
| Whole number | 107 | 169 | 276 |
| Discharged | 26 | 46 | 72 |
| Remaining, November 30, 1850 | 81 | 123 | 204 |
| Of those discharged, there were cured | 12 | 25 | 37 |
| Died | 8 | 17 | 25 |

Died of consumption 4, general debility 3, paralysis 2, exhaustion 2, dropsy 1, erysipelas 1, marasmus 1, dysentery 11.

"Dysentery has been the most prevalent and fatal disease with which we have had to contend. It commenced about the 1st of August, and continued till about the 1st of November. During this period 47 cases occurred of a severe and very intractable character. Of this number, 10 died. It proved most fatal among the aged and the melancholic, and took off but one case in which there was any certainty of recovery from mental disease. It was remarked that only the most emaciated, or such as were suffering from other and long standing disease, succumbed to dysentery; the fat and more robust who were attacked survived. One patient, an Indian, in good bodily health, afflicted with chronic mania, and who had been insane three years, was seized with the severest form of dysentery which has ever come under my observation. While in the height of the malady, his mental operations began to undergo a change, after which his mental and bodily convalescence went on together, and resulted in the perfect restoration of the entire man. Another, a man who had been insane over twenty years, and quite a difficult one to manage, owing to his strong mischievous propensities, was attacked with the same affection, and remained dangerously ill for some weeks. He recovered from dysentery, and now no patient in the house is more quiet and controllable. Indeed, to many he would appear mentally sound."

In June, 1851, Dr. Stedman resigned his office, and Dr. Clement A. Walker was elected as his successor. The report for that year is from the pen of the latter.

| | Men. | Women. | Total. |
|---|---|---|---|
| Patients, November 20, 1850 | 81 | 123 | 204 |
| Admitted in the course of the year | 46 | 46 | 92 |
| Whole number | 127 | 169 | 296 |
| Discharged | 27 | 28 | 55 |
| Remaining, November 30, 1851 | 100 | 141 | 241 |
| Of those discharged, there were cured | 13 | 14 | 27 |
| Died | 10 | 12 | 22 |

*Causes of death.* — Consumption 3, exhaustion 3, general debility 2, dysentery 2, epilepsy 2, marasmus 2, apoplexy, typhoid fever, typhus fever, pneumonia, inflammation of bladder, general paralysis, and chronic diarrhœa, 1 each.

"But one epidemic has appeared among us the past year, and although dysentery was prevalent, numbering nearly fifty cases, yet it was of mild type, and was fatal in but one or two instances."

The following case of "an intelligent Irish lad" is worthy of a place upon permanent record:—

"The little fellow, but thirteen years of age, arrived at Boston, on board an emigrant vessel, in July last (1851), having no friends here, with the exception of a brother, who had preceded him but a few months. He landed on Thursday, and on Saturday became a raving maniac. Confused by the strangeness, and, to his eyes, the magnificence of the city, which, for weeks, had been the culminating point of his anticipations, he wandered about, gazing upon the novelties by day, and dreaming of them by night, until he believed himself the inhabitant of a fairy land, and could not recognise the brother whose bed he shared; 'for,' said he, 'he was dressed so nice, and we usedn't to be so at home.' Reason soon fled, and for weeks he by turns babbled like a child and raved like a madman. At length convalescence was established, and has since rapidly progressed. A few weeks more and he will doubtless go out from us whole."

Of the 92 patients admitted in the course of the year, 61 were foreigners, 50 of them from Ireland.

Persons acquainted with the subject will perceive, by the statistics herein quoted, how, as time has progressed, the great predominance in the number of males over that of females in our public institutions for the insane has disappeared. Indeed, *from these data*, it would appear that there are more insane females than males. Excluding the Maine Hospital, where, since the conflagration, the apartments for men greatly exceed those for women, the number of patients remaining in the above-mentioned asylums, at the time of last report (except the McLean, of which the latest report does not distinguish the numbers of the sexes) was, of males 582, females 645, and the number of admissions, in course of the preceding year, males 367, females 399.—(*American Journal of Medical Science, for January*, 1854.)

